# Association of Urinary Potassium Excretion with Blood Pressure Variability and Cardiovascular Outcomes in Patients with Pre-Dialysis Chronic Kidney Disease

**DOI:** 10.3390/nu13124443

**Published:** 2021-12-13

**Authors:** Sang Heon Suh, Su Hyun Song, Tae Ryom Oh, Hong Sang Choi, Chang Seong Kim, Eun Hui Bae, Kook-Hwan Oh, Joongyub Lee, Seung Hyeok Han, Yeong Hoon Kim, Dong-Wan Chae, Seong Kwon Ma, Soo Wan Kim, on behalf of the Korean Cohort Study for Outcomes in Patients with Chronic Kidney Disease (KNOW-CKD) Investigators

**Affiliations:** 1 Department of Internal Medicine, Chonnam National University Medical School and Chonnam National University Hospital, Gwangju 61496, Korea; medssh1984@gmail.com (S.H.S.); sudang_@naver.com (S.H.S.); tryeomoh@hanmail.net (T.R.O.); hongsang38@hanmail.net (H.S.C.); laminion@hanmail.net (C.S.K.); baedak76@gmail.com (E.H.B.); 2 Department of Internal Medicine, Seoul National University Hospital, Seoul 06591, Korea; ohchris@hanmail.net; 3 Department of Prevention and Management, School of Medicine, Inha University, Incheon 22212, Korea; tp240@naver.com; 4 Department of Internal Medicine, College of Medicine, Institute of Kidney Disease Research, Yonsei University, Seoul 03722, Korea; hansh@yuhs.ac; 5 Department of Nephrology, College of Medicine, Inje University, Busan 47392, Korea; yeonghnl@inje.ac.kr; 6 Department of Internal Medicine, Seoul National University Bundang Hospital, Seongnam 13620, Korea; cdw1302@snubh.org

**Keywords:** blood pressure variability, chronic kidney disease, dietary potassium intake, extended major cardiovascular event, urine potassium

## Abstract

Dietary potassium intake is a dilemma in patients with chronic kidney disease (CKD). We investigated the association of urine potassium excretion, a surrogate for dietary potassium intake, with blood pressure variability (BPV) and cardiovascular (CV) outcomes in patients with pre-dialysis CKD. A total of 1860 participants from a cohort of pre-dialysis CKD (KNOW-CKD) patients were divided into the quartiles by spot urine potassium-to-creatinine ratio. The first quartile (26.423 ± 5.731 mmol/gCr) was defined as low urine potassium excretion. Multivariate linear regression analyses revealed an independent association of low urine potassium excretion with high BPV (adjusted β coefficient 1.163, 95% confidence interval 0.424 to 1.901). Cox regression analyses demonstrated that, compared to high urine potassium excretion, low urine potassium excretion is associated with increased risk of CV events (adjusted hazard ratio 2.502, 95% confidence interval 1.162 to 5.387) but not with all-cause mortality. In conclusion, low urine potassium excretion is associated with high BPV and increased risk of CV events in patients with pre-dialysis CKD. The restriction of dietary potassium intake should be individualized in patients with pre-dialysis CKD.

## 1. Introduction

Blood pressure (BP) variability (BPV) is an emerging mediator of clinical outcomes [1,2,3]. The association between long-term BPV and the risk of incident chronic kidney disease (CKD) has been repeatedly reported in the general population [4,5,6]. In patients with CKD, higher long-term BPV is associated with more rapid progression of CKD [7], poor cardiovascular (CV) outcomes and increased all-cause mortality [8]. A close relationship between BPV and the amount of dietary sodium has been reported both in normotensive [9] and hypertensive [10] subjects. Conversely, randomized trials proved that oral potassium supplements significantly reduce systolic BP (SBP), but not diastolic BP (DBP) [11], and improve arterial compliance [12] in the general population. Yet, the association between dietary potassium intake and BPV has not been elucidated specifically in CKD patients.

It is well known that high sodium intake not only increases blood pressure but also plays a role in endothelial dysfunction, CV structure and function, albuminuria and CKD progression, and CV mortality in the general population. Conversely, dietary potassium intake attenuates the effects mediated by dietary sodium intake [13,14]. However, dietary potassium intake is a dilemma in patients with CKD. Excess potassium intake imposes a potential risk of fatal arrhythmia due the limited capacity of potassium excretion in patients with advanced CKD [15], even though the restriction of dietary potassium intake in this population has been rarely advocated by supporting data. Rather, mounting evidence now suggests the benefits of high potassium diets in CKD patients. A cohort study composed of CKD stages 2–4 patients in the United States reported that high urine potassium excretion, a surrogate for dietary potassium intake, is associated with an increased risk of all-cause mortality [16]. Another cohort study of non-dialysis-dependent CKD patients revealed that high urine potassium excretion is associated with better composite renal outcomes [17]. It remains unclear, however, whether low dietary potassium negatively affects CV outcomes in CKD patients, while potassium-rich diets reduce CV events and all-cause mortality in the general population [18,19,20,21].

In the present study, using potassium-to-creatinine ratio (K^+^/Cr) in spot urine as a surrogate for dietary potassium intake, we investigated the association of low urine potassium with visit-to-visit long-term BPV in patients with CKD. We also examined whether other urinary electrolyte parameters, such as sodium-to-creatinine ratio (Na^+^/Cr) and sodium-to-potassium ratio (Na^+^/K^+^), are significantly related to BPV. Importantly, the impact of low urine potassium on the CV outcomes in CKD patients was analyzed.

## 2. Materials and Methods

### 2.1. Study Design and Participants

**Figure 1:**
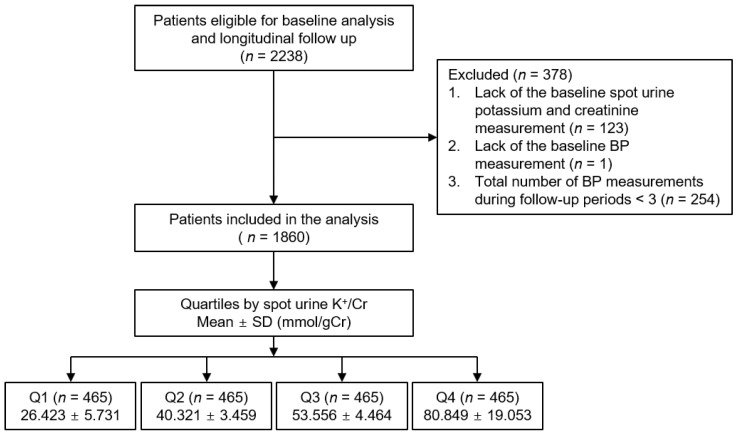
Flow diagram of the study participants. Abbreviations: Cr, creatinine; K^+^/Cr, potassium/creatinine ratio; SD, standard deviation; Q1, 1st quartile; Q2, 2nd quartile; Q3, 3rd quartile; Q4, 4th quartile.

The Korean Cohort Study for Outcomes in Patients with Chronic Kidney Disease (KNOW-CKD) is a nationwide prospective cohort study involving 9 tertiary-care general hospitals in Korea [22]. Korean patients with CKD from stage 1 to pre-dialysis stage 5, who voluntarily provided informed consent, were enrolled between 2011 and 2015. The study was conducted in accordance with the principles of the Declaration of Helsinki, and the study protocol was approved by the institutional review boards of participating centers, including Seoul National University Hospital, Yonsei University Severance Hospital, Kangbuk Samsung Medical Center, Seoul St. Mary’s Hospital, Gil Hospital, Eulji General Hospital, Chonnam National University Hospital, and Pusan Paik Hospital. All participants had been under close observation, and participants who experienced study outcomes were reported by each participating center. The study observation period ended on 31 March 2019. A total of 2238 subjects were longitudinally followed (Figure 1). After further excluding those lacking the baseline spot urine potassium and creatinine measurement, lacking the baseline BP measurement, or with a total number of BP measurements during the follow-up period of less than 3, 1860 subjects were finally included for the analyses. The median follow-up duration was 5.623 years.

### 2.2. Data Collection

Demographic information was collected from all eligible participants, including age, gender, smoking history, medications (angiotensin-converting enzyme inhibitors and angiotensin receptor blockers (ACEi/ARBs), diuretics, and statins), and comorbid conditions, at the time of screening. Anthropometric indices (height, weight circumference (WC), and systolic and diastolic blood pressures (SBP and DBP)) were also measured. Body mass index (BMI) was calculated as weight/height^2^ (kg/m^2^). Laboratory data included hemoglobin, creatinine, albumin, glucose, triglyceride (TG), total cholesterol, low-density lipoprotein cholesterol, high-density lipoprotein cholesterol (HDL-C), and high sensitive C-reactive protein (hs-CRP). Serum creatinine was measured by an isotope dilution mass spectrometry–traceable method, and estimated glomerular filtration rate (eGFR) was calculated using the Chronic Kidney Disease Epidemiology Collaboration (CKD-EPI) equation [23]. CKD stages were determined by the Kidney Disease Improving Global Outcomes guidelines [24]. Urinary metrics, such as sodium, potassium, and creatinine (Cr), were measured in random, preferably second-voided, spot urine samples at the baseline. To measure the urinary potassium excretion, 24 h urine samples were collected in a total of 843 subjects. Subjects were divided into quartiles (Q1 to Q4) by spot urine K^+^/Cr (Figure 1 and Table 1), Na^+^/Cr (Supplemental Table S1), or Na^+^/K^+^ (Supplemental Table S2), where the 1st and 4th quartiles were defined as low and high, respectively.

### 2.3. Determination of Visit-to-Visit BPV

BP was measured by an electronic sphygmomanometer after seated rest for 5 min, at 0, 6, and 12 months and then yearly thereafter up to 8 years. Long-term visit-to-visit BPV was determined by average real variability (ARV), standard deviation (SD), and coefficient of variation (CoV) of SBP across visits. The median number of BP measurements in the study participants was 6.

### 2.4. Study Outcomes

The outcomes of interest were extended major cardiovascular events (eMACEs) and all-cause mortality. eMACE was defined as the first occurrence of cardiac death and nonfatal CV events, including any nonfatal coronary artery event (unstable angina, myocardial infarction, or coronary intervention/surgery), hospitalization for heart failure, ischemic or hemorrhagic stroke, or symptomatic arrhythmia.

### 2.5. Statistical Analysis

Continuous variables were expressed as mean ± standard deviation or median (interquartile range). Categorical variables were expressed as number of participants and percentage. For descriptive analyses, Student’s T-test or one-way analysis of variance and χ2 test were used for continuous and categorical variates, respectively. The participants with any missing data were excluded for further analyses. The multivariate linear regression model was adjusted for covariates to address the association between low urine potassium excretion and visit-to-visit BPV. The models were adjusted for age, sex, Charlson comorbidity index, history of diabetes mellitus (DM), BMI, WC, SBP, DBP, medication (ACEi/ARBs, diuretics, number of antihypertensive drugs), hemoglobin, albumin, fasting glucose, HDL-C, TG, 25(OH) vitamin D, hs-CRP levels, eGFR, and spot urine albumin-to-creatinine ratio (ACR). The results of multivariate linear regression models were presented as β coefficient and 95% CIs. Survival time was defined as the interval between the enrollment and the first occurrence of the outcomes. Patients lost to follow-up were censored at the date of the last visit. To assess the association between low urine potassium excretion and the outcomes, Cox proportional hazard regression models were analyzed. The models were adjusted for age, sex, Charlson comorbidity index, history of DM, BMI, WC, SBP, DBP, medication (ACEi/ARBs, diuretics, number of antihypertensive drugs), hemoglobin, albumin, fasting glucose, HDL-C, TG, 25(OH) vitamin D, hs-CRP levels, eGFR, spot urine ACR, and ARV of SBP. The results of Cox proportional hazard models were presented as hazard ratios (HRs) and 95% confidence intervals (CIs). To confirm our findings, we conducted sensitivity analyses. To eliminate the possibility that urine potassium excretion may not reflect dietary potassium intake proportionally in patients with advanced CKD, those with CKD stage 5 at the baseline (*n* = 70) were excluded in multivariate linear regression and Cox regression analyses. In addition, as the subjects with eGFR ≥ 90 mL/min/1.73 m^2^ were considered close to normal kidney function, we excluded the subjects with eGFR ≥ 90 mL/min/1.73 m^2^ (*n* = 213) and conducted multivariate linear regression and Cox regression analyses. Two-sided *p* values < 0.05 were considered statistically significant. Statistical analysis was performed using SPSS for Windows version 22.0 (IBM Corp., Armonk, NY, USA) and R (version 4.1.1; R project for Statistical Computing, Vienna, Austria).

## 3. Results

### 3.1. Baseline Characteristics

To clarify the baseline characteristics of study participants, the subjects were divided into quartiles by spot urine K^+^/Cr (Table 1). The follow-up durations were not different among the quartile groups. As urine potassium excretion increased from the 1st quartile (Q1) to the 4th quartile (Q4), the mean age significantly increased, while the frequency of male participants decreased. The Charlson comorbidity index, which quantifies the burden of comorbid conditions, was marginally lower in Q4 than in Q1. The frequency of diuretic use and medication of antihypertensive drugs no less than three was significantly higher in Q1. The other demographic and anthropometric findings were not significantly different among the groups. Serum potassium levels were not different across the quartile groups, while 24 h urine potassium proportionally increased from Q1 to Q4. Total cholesterol, HDL-C, and low-density lipoprotein cholesterol were lowest and highest in Q1 and Q4, respectively. Serum 25(OH) vitamin D level increased as the urine potassium excretion increased. Serum hsCRP level significantly differed among the groups, with the highest in Q2 and the lowest in Q4. Whereas no significant difference in spot urine ACR was observed across the groups, eGFR tended to be preserved as urine potassium excretion increased. Accordingly, those with early stages of CKD were relatively abundant in Q4, while those with advanced stages of CKD were more frequently observed in Q1.

### 3.2. Association between Spot Urine K^+^/Cr and BPV in Patients with Pre-Dialysis CKD

**Figure 2:**
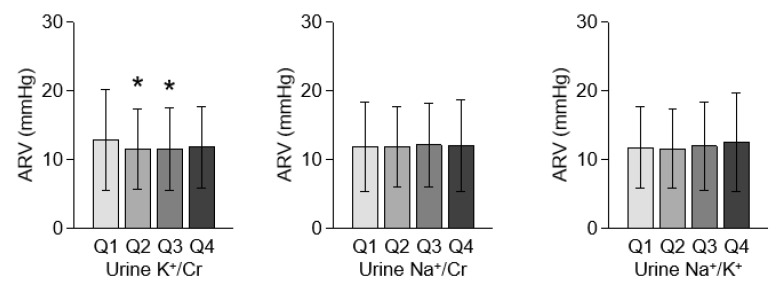
Comparison of BP variability in the quartiles by urine K^+^/Cr. Error bars indicate standard deviation. *, *p* < 0.05 versus Q1 by one-way ANOVA with Scheffe’s post hoc analyses. Abbreviations: ARV, average real variability; BP, blood pressure; BPV, blood pressure variability; K^+^/Cr, potassium-to-creatinine ratio; Na^+^/Cr, sodium-to-creatinine ratio; Na^+^/K^+^, sodium-to-potassium ratio; Q1, 1st quartile; Q2, 2nd quartile; Q3, 3rd quartile; Q4, 4th quartile.

To determine the association between urine potassium excretion and BPV, BPV was compared in the quartiles by urine K^+^/Cr (Figure 2). The ARV of Q1 (i.e., low urine potassium excretion, spot urine K^+^/Cr ≤ 34.209 mmol/g) was greater than that of the rest, although statistical significance was not observed between Q1 and Q4. ARV was also compared in the quartiles by spot urine Na^+^/Cr and Na^+^/K^+^, which failed to reveal any significant differences across the groups.

To examine whether low urine potassium excretion is independently associated with greater BPV, a multivariate linear regression model was analyzed with covariate adjustment (Table 2). In the analysis including all subjects, low urine K^+^/Cr was independently associated with ARV (adjusted β coefficient 1.163, 95% CI 0.424 to 1.901), but not with SD (adjusted β coefficient 0.431, 95% CI −0.176 to 1.037) or CoV (adjusted β coefficient 0.004, 95% CI −0.001 to 0.009). None of high urine K^+^/Cr, low urine Na^+^/Cr, high Na^+^/Cr, low urine Na^+^/K^+^, or high urine Na^+^/K^+^ was significantly associated with any BPV indices.

### 3.3. Association of Low Urine Potassium Excretion with Adverse CV Outcomes in Patients with Pre-Dialysis CKD

To investigate the association between low urine potassium excretion and clinical outcomes, a Cox regression model was analyzed with covariate adjustment (Table 3). Compared to high urine potassium excretion, low potassium excretion was associated with increased risk of eMACE (adjusted HR 2.502, 95% CI 1.162 to 5.387). Low urine potassium excretion was not associated with all-cause mortality (adjusted HR 0.604, 95% CI 0.204 to 1.519). Urine sodium excretion (Supplemental Table S3) and urine Na^+^/K^+^ (Supplemental Table S4) were not significantly associated with eMACE or all-cause mortality, suggesting that potassium excretion level might better predict the CV outcomes in patients with CKD using spot urine samples.

### 3.4. Sensitivity Analyses

To eliminate the possibility that urine potassium excretion may not reflect dietary potassium intake proportionally in patients with advanced CKD, those with CKD stage 5 at the baseline (*n* = 70) were excluded in multivariate linear regression (Supplemental Table S5) and Cox regression analyses (Supplemental Table S6). Low urine K^+^/Cr was robustly associated with an increase in ARV (Adjusted β coefficient 1.092, 95% CI 0.353 to 1.832) and was also associated with increased risk of eMACE (adjusted HR 2.475, 95% CI 1.128 to 5.428). After excluding the subjects with eGFR ≥ 90 mL/min/1.73 m^2^, who were considered close to normal kidney function, multivariate linear regression analysis (Supplemental Table S7) revealed that low urine K^+^/Cr is still associated with an increase in ARV (Adjusted β coefficient 1.225, 95% CI 0.433 to 2.018). Low urine K^+^/Cr was also robustly associated with increased risk of eMACE (adjusted HR 2.857, 95% CI 1.247 to 6.546), even after excluding 70 participants who were CKD stage 1 patients at the baseline (Supplemental Table S8).

### 3.5. Subgroup Analyses

To evaluate whether the association of low urine potassium excretion with BPV and the risk of CV events is modified by subgroups, we conducted subgroup analyses. The subgroups were stratified by age (<60 or ≥60 years), diuretic use (without or with), eGFR (≥45 or <45 mL/min/1.73 m^2^), and spot urine ACR (<300 or ≥300 mg/g). Multivariate linear regression analysis demonstrated that the association of low urine potassium excretion with an increase in BPV is significantly more prominent in the subjects with <45 mL/min/1.73 m^2^ than in the subjects with ≥45 mL/min/1.73 m^2^ (*p* for interaction = 0.026) (Supplemental Table S9). Cox regression analysis regarding the association of low urine potassium excretion with the risk of eMACE (Table 4) and all-cause mortality (Supplemental Table S10) revealed that *P* for interactions was >0.05 for all subgroups, suggesting that the association of low urine potassium excretion with increased risk of eMACE or all-cause mortality is not modified by these factors.

## 4. Discussion

In the present study, we demonstrated that low urine potassium excretion is independently associated with high BPV in patients with pre-dialysis CKD. We also proved that low urine potassium excretion is significantly associated with increased risk of eMACE. Subgroup analyses demonstrated that, while the association between low urine potassium excretion and the risk of eMACE was not modified by age, diuretic use, eGFR, and spot urine ACR, the association of low urine potassium excretion with high ARV is more prominent in subjects with eGFR < 45 mL/min/1.73 m^2^.

Despite the concern that excess potassium intake may impose a potential risk of fatal arrhythmia due to hyperkalemia in patients with advanced CKD [15], recent studies have provided evidence that the restriction of dietary potassium intake may attenuate the beneficial action of potassium with respect to CKD progression [17] and all-cause mortality [16]. Our data further expanded the advantage conferred by dietary potassium intake to the CV outcomes in CKD patients, which is largely in line with the findings observed in the general population [18,19,20,21]. The precise mechanism for how dietary potassium intake improves CV outcomes in CKD patients as well as in the general population is still unclear. Although potassium, as a potent vasodilator, reduces vascular resistance [25,26], and thereby its supplementation leads to a decrease in BP [21], the measured SBP of the subjects in the current study was not concordant with urine potassium excretion, as SBP was higher in the 3rd and 4th quartiles. We therefore hypothesize that stabilization of BPV, instead of mean BP control, may mediate the impact of dietary potassium intake on the improvement of clinical outcomes, such as eMACE.

The independent role of BPV has been illustrated by several reports [4,5,6,7,16,27]. Although the underlying mechanism of increased BPV has not yet been elucidated, evidence so far suggests certain biological processes may drive the worsening of BPV. For instance, decreased arterial elasticity and increased arterial stiffness have been observed in those with greater BPV [28,29]. Impairment of nitric oxide-drive vascular relaxation [30] may also partly contribute to the increase of BPV. A previous study reported that markers of vascular inflammation are associated with high BPV, independent of SBP [31]. Considering the theoretical action of potassium ion in vascular beds [32], it is reasonable to expect the role of dietary potassium intake in the stabilization of BPV. In this context, our data present a clinical relevance that, in addition to dietary sodium restriction [9,10], dietary potassium intake may modulate BPV in patients with CKD.

Nevertheless, it is still unknown whether a potassium-rich diet or a specified dietary potassium intake should be encouraged in patients with CKD, considering that the association of low urine potassium excretion with high ARV was more prominent in subjects with eGFR < 45 mL/min/1.73 m^2^ (Supplementary Table S7). Taken together, the studies thus far lack evidence that dietary potassium restriction reduces the risk of CKD progression, CV events, or all-cause mortality. It seems prudent that the restriction of dietary potassium intake should be individualized for CKD patients, even when their CKD stages are advanced, provided that serum potassium level and electrocardiogram are closely monitored [33].

The definition of BPV is variable [34]. Very short-term BPV is calculated from the measurement of beat-to-beat BPs over variable time periods. Short-term to mid-term BPV is defined from the BP measurement by ambulatory or home BP monitoring, whereas visit-to-visit BPV is obtained from a long-term measurement of BP via office or ambulatory BP monitoring. Even a visit-to-visit BPV via office BP monitoring has been variously defined. The SD of SBP and/or DBP measurements across visits was adopted in some studies [3,7], CoV (calculated as SD divided by the mean BP) in other studies [5,29,35], and both in other studies [4,6,36]. SD is relatively easier and probably more practical, but tends to correlate with the average of blood pressure measurements. Therefore, CoV, which is calculated by dividing the mean value by the SD, has been also used to determine visit-to-visit BPV [37]. ARV, which is defined as the average of the absolute differences of consecutive measurements, is a more reliable and sensitive representation of time series variability, despite relatively low sampling frequency compared to SD [38]; this is the reason why ARV was used for the primary analysis in the current study. The other index of BPV is the variation independent of the mean (VIM), which is calculated based on non-linear regression [39]. However, VIM is considered to be a better index of BP variability than the other indices, because VIM is not associated with mean blood pressure. However, VIM was not evaluated in this study, as there is a significant difference between VIM and the other indices of BPV (SD, CoV, and ARV) and as it is less practical in clinical perspectives [37].

There are a number of limitations in this study. First, we are not able to clarify the casual relation between low urine potassium excretion and the risk of eMACE, primarily due to the observational nature of the current study. Second, despite the robust relation between low urine potassium excretion and poor CV outcomes, all-cause mortality was not significantly associated with low urine potassium excretion. This could be primarily attributed to the relatively rare frequency of death events, since the events occurred only in 71 out of 1860 subjects (3.8%). We should mention that this may be changeable, as the death events accumulate during the follow up. Third, although we adopted K^+^/Cr in spot urine samples as a surrogate for dietary potassium intake, 24 h urinary potassium excretion is still a gold standard for estimating daily potassium intake. Yet, the urine collection for 24 h imposed a substantial burden to the subjects, and 24 h urine potassium could be measured only for a portion of the participants (843 out of 1860). Thus, the estimation of dietary potassium intake from spot urine K^+^/Cr seems a realistic and practical alternative. Fourth, urinary potassium excretion may not reflect dietary potassium intake accurately in the subjects with advanced CKD, due to the fact that the contribution of the gut in potassium elimination steadily increases with the decreasing GFR. However, we conducted a sensitivity analysis that excluded the subjects with CKD stage 5 at the baseline, to minimize the possibility that urine potassium excretion may not reflect dietary potassium intake proportionally in patients with advanced CKD. Fifth, it is possible that the risk of sever hyperkalemia could be underestimated, as the events due to severe hyperkalemia were not clearly defined; however, we included symptomatic arrhythmia as a component of eMACE. Sixth, as the overall nutritional status at the baseline seems better in the subjects with higher urinary potassium excretion, it is still possible that the regression analyses may not provide sufficient adjustment to compensate for the differences shown in the baseline characteristics. Seventh, despite the association between urine potassium excretion and BPV in this study, we were not able to determine the precise mechanism for how urine potassium, but not sodium, excretion is specifically associated with BPV in subjects with pre-dialysis CKD. Lastly, as this cohort study enrolled only Koreans, caution is required to extrapolate the data in the present study to other populations.

In conclusion, we report that low urine potassium excretion is independently associated with high BPV and is also significantly associated with increased risk of eMACE in patients with pre-dialysis CKD. Considering the potential CV benefit conferred by a high potassium diet, the restriction of dietary potassium intake should be individualized in patients with pre-dialysis CKD.

## Figures and Tables

**Table 1:** Baseline characteristics of study participants in the quartiles by spot urine K^+^/Cr.

	Spot Urine K^+^/Cr				*p* Value
	Q1	Q2	Q3	Q4	
Follow-up duration (year)	5.150 ± 1.766	5.237 ± 1.724	5.180 ± 1.715	5.180 ± 1.709	0.892
Age (year)	50.785 ± 13.566	53.880 ± 11.609	54.487 ± 11.157	55.282 ± 11.198	<0.001
Male	358 (77.0)	330 (71.0)	260 (55.8)	172 (37.1)	<0.001
Charlson comorbidity index					0.049
0–3	316 (68.0)	348 (74.8)	353 (75.9)	354 (76.3)	
4–5	137 (29.5)	110 (23.7)	108 (23.2)	105 (22.6)	
≥6	12 (2.6)	7 (1.5)	5 (1.1)	5 (1.1)	
Primary renal disease					0.218
DM	128 (27.5)	106 (22.8)	89 (19.1)	105 (22.6)	
HTN	95 (20.4)	102 (21.9)	89 (19.1)	92 (19.8)	
GN	154 (33.1)	144 (31.0)	164 (35.2)	152 (32.8)	
TID	3 (0.6)	4 (0.9)	3 (0.6)	4 (0.9)	
PKD	56 (12.0)	81 (17.4)	91 (19.5)	84 (18.1)	
Others	29 (6.2)	28 (6.0)	30 (6.4)	27 (5.8)	
History of DM	164 (35.3)	144 (31.0)	126 (27.0)	156 (33.6)	0.060
Medication					
ACEi/ARBs	399 (89.9)	382 (89.7)	415 (94.1)	395 (90.2)	0.068
Diuretics	169 (38.1)	121 (27.7)	140 (31.7)	131 (29.9)	0.007
Number of antihypertensive drugs ≥ 3	165 (35.5)	144 (31.0)	135 (29.0)	121 (26.1)	0.016
BMI (kg/m^2^)	24.635 ± 3.496	24.552 ± 3.402	24.555 ± 3.196	24.517 ± 3.403	0.960
WC (cm)	87.461 ± 9.775	88.195 ± 10.083	86.987 ± 9.343	86.682 ± 9.475	0.111
SBP (mmHg)	127.194 ± 16.501	127.860 ± 15.620	126.863 ± 14.774	126.170 ± 14.620	0.407
DBP (mmHg)	76.428 ± 11.306	77.204 ± 11.317	77.485 ± 11.010	76.069 ± 9.963	0.165
Laboratory findings					
Serum K^+^ (mEq/L)	4.594 ± 0.682	4.547 ± 0.661	4.529 ± 0.631	4.555 ± 0.649	0.511
24 h urine K^+^ (mEq/day)	42.027 ± 17.588	51.258 ± 43.036	56.539 ± 19.539	63.575 ± 23.118	<0.001
Hemoglobin (g/dL)	12.910 ± 2.116	13.138 ± 1.995	13.027 ± 1.947	12.835 ± 1.816	0.098
Albumin (g/dL)	4.192 ± 0.388	4.199 ± 0.412	4.189 ± 0.370	4.245 ± 0.360	0.085
Total cholesterol (mg/dL)	168.254 ± 39.284	172.600 ± 38.473	177.301 ± 37.889	177.711 ± 34.974	<0.001
HDL-C (mg/dL)	46.447 ± 15.762	48.837 ± 15.353	50.428 ± 14.616	52.714 ± 15.800	<0.001
LDL-C (mg/dL)	93.485 ± 31.973	94.838 ± 30.550	97.863 ± 28.925	99.501 ± 29.626	0.011
TG (mg/dL)	163.899 ± 104.737	158.026 ± 96.706	155.236 ± 101.332	150.296 ± 91.559	0.213
Fasting glucose (mg/dL)	112.513 ± 46.816	109.218 ± 36.002	107.429 ± 33.016	110.770 ± 35.886	0.217
25(OH) Vitamin D (ng/mL)	17.190 ± 9.218	18.653 ± 9.408	18.806 ± 9.916	19.683 ± 10.427	0.017
hsCRP (mg/dL)	0.600 [0.100, 1.860]	0.780 [0.100, 1.800]	0.550 [0.200, 1.400]	0.500 [0.200, 1.600]	0.004
Spot urine ACR (mg/gCr)	349.855 [103.771, 1050.062]	280.683 [57.545, 856.995]	318.405 [71.818, 901.375]	306.642 [48.397, 801.223]	0.387
eGFR (mL/min/1.73 m^2^)	48.048 ± 28.928	51.516 ± 27.605	56.207 ± 58.677	64.745 ± 31.792	<0.001
CKD stages					<0.001
Stage 1	49 (10.5)	62 (13.3)	73 (15.7)	129 (27.8)	
Stage 2	78 (16.8)	84 (18.1)	107 (23.0)	103 (22.2)	
Stage 3a	73 (15.7)	87 (18.7)	85 (18.2)	77 (16.6)	
Stage 3b	116 (24.9)	118 (25.4)	107 (23.0)	81 (17.5)	
Stage 4	129 (27.7)	96 (20.6)	78 (16.7)	62 (13.4)	
Stage 5	20 (4.3)	18 (3.9)	16 (3.4)	12 (2.6)	

Values for categorical variables are given as number (percentage); values for continuous variables, as mean ± standard deviation or median [interquartile range]. Abbreviations: ACEi, angiotensin converting enzyme inhibitor; ACR, albumin-to-creatinine ratio; ARB, angiotensin receptor blocker; BMI, body mass index; CKD, chronic kidney disease; CKD-EPI, Chronic Kidney Disease Epidemiology Collaboration; Cr, creatinine; DBP, diastolic blood pressure; DM, diabetes mellitus; eGFR, estimated glomerular filtration rate; hsCRP, high-sensitivity C-reactive protein; HTN, hypertension; K^+^/Cr, potassium/creatinine ratio; SBP, blood pressure; WC, waist circumference; WHR, waist-to-hip ratio.

**Table 2:** Multivariate linear regression analysis for BPV with various electrolyte profiles in spot urine samples.

	Unadjusted		Adjusted	
	Coefficients (95% CIs)	*p* Value	Coefficients (95% CIs)	*p* Value
Low urine K^+^/Cr				
ARV	1.260 (0.545, 1.975)	0.001	1.163 (0.424, 1.901)	0.002
SD	0.511 (−0.071, 1.094)	0.085	0.431 (−0.176, 1.037)	0.164
CoV	0.005 (0.000, 0.009)	0.057	0.004 (−0.001, 0.009)	0.138
High urine K^+^/Cr				
ARV	−0.130 (−0.857, 0.598)	0.727	0.127 (−0.634, 0.887)	0.744
SD	0.261 (−0.330, 0.853)	0.386	0.468 (−0.154, 1.091)	0.140
CoV	0.002 (−0.002, 0.007)	0.299	0.004 (−0.001, 0.009)	0.101
Low urine Na^+^/Cr				
ARV	−0.139 (−0.871, 0.593)	0.710	0.169 (−0.547, 0.884)	0.644
SD	−0.257 (−0.851, 0.338)	0.397	−0.018 (−0.605, 0.568)	0.951
CoV	−0.001 (0.006, 0.004)	0.611	0.000 (−0.005, 0.005)	0.978
High urine Na^+^/Cr				
ARV	0.105 (−0.614, 0.824)	0.774	−0.190 (−0.904, 0.524)	0.602
SD	0.127 (−0.457, 0.711)	0.669	−0.116 (−0.701, 0.469)	0.698
CoV	0.000 (−0.005, 0.004)	0.915	−0.001 (−0.006, 0.004)	0.659
Low urine Na^+^/K^+^				
ARV	−0.176 (−0.906, 0.553)	0.635	0.222 (−0.483, 0.927)	0.536
SD	0.060 (−0.533, 0.652)	0.844	0.343 (−0.235, 0.920)	0.245
CoV	0.001 (−0.004, 0.006)	0.654	0.003 (−0.002, 0.007)	0.263
High urine Na^+^/K^+^				
ARV	0.873 (0.158, 1.588)	0.017	0.275 (−0.426, 0.976)	0.442
SD	0.545 (−0.036, 1.126)	0.066	0.146 (−0.429, 0.720)	0.618
CoV	0.003 (−0.002, 0.008)	0.220	0.001 (−0.004, 0.005)	0.722

Models were adjusted for age, sex, Charlson comorbidity index, history of DM, medication (ACEi/ARBs, diuretics, number of antihypertensive drugs), BMI, WC, SBP, DBP, hemoglobin, albumin, fasting serum glucose, HDL-C, TG, 25(OH) vitamin D, hs-CRP levels, eGFR, and spot urine ACR. Abbreviations: ARV, average real variability; CI, confidence interval; CoV, coefficient of variation; K^+^/Cr, potassium-to-creatinine ratio; Na^+^/Cr, sodium-to-creatinine ratio; Na^+^/K^+^, sodium-to-potassium ratio; SD, standard deviation.

**Table 3:** Cox regression analysis of urine potassium excretion for clinical outcomes.

	Spot Urine K^+^/Cr	Cases, *n* (%)	Unadjusted		Adjusted	
			HR (95% CIs)	*p* Value	HR (95% CIs)	*p* Value
eMACE	Q1	36 (7.7)	1.899 (1.114, 3.239)	0.018	2.502 (1.162, 5.387)	0.019
	Q2	27 (5.8)	1.727 (0.992, 3.006)	0.053	1.120 (0.512, 2.451)	0.777
	Q3	29 (6.2)	1.393 (0.812, 2.389)	0.228	1.590 (0.777, 3.252)	0.204
	Q4	40 (8.6)	Reference		Reference	
All-cause mortality	Q1	17 (3.7)	0.733 (0.321, 1.672)	0.460	0.604 (0.240, 1.519)	0.284
	Q2	20 (4.30)	1.406 (0.694, 2.846)	0.344	1.222 (0.560, 2.668)	0.615
	Q3	17(3.6)	1.037 (0.487, 2.207)	0.925	0.953 (0.433, 2.099)	0.905
	Q4	17 (3.7)	Reference		Reference	

Models were adjusted for age, sex, Charlson comorbidity index, history of DM, medication (ACEi/ARBs, diuretics, number of antihypertensive drugs), BMI, WC, SBP, DBP, hemoglobin, albumin, fasting serum glucose, HDL-C, TG, 25(OH) vitamin D, hs-CRP levels, eGFR, spot urine ACR, and ARV. Abbreviations: CI, confidence interval; K^+^/Cr, potassium-to-creatinine ratio; CI, confidence interval; eGFR, estimated glomerular filtration rate; HR, hazard ratio; Q1, 1st quartile; Q2, 2nd quartile; Q3, 3rd quartile; Q4, 4th quartile.

**Table 4:** Cox regression analysis of urine potassium excretion for eMACE in various subgroups.

	Spot Urine K^+^/Cr	Cases, *n* (%)	Unadjusted		Adjusted	
			HR (95% CIs)	*p* for Interaction	HR (95% CIs)	*p* for Interaction
Age < 60 years	Q1	14 (4.3)	4.162 (1.523, 11.373)	0.672	0.502 (0.202, 12.797)	0.780
	Q2	11 (3.5)	3.800 (1.334, 10.826)		5.681 (0.378, 85.486)	
	Q3	16 (5.3)	4.155 (1.492, 11.570)		2.271 (0.203, 25.433)	
	Q4	13 (4.6)	Reference		Reference	
Age ≥ 60 years	Q1	22 (15.4)	1.221 (0.631, 2.365)		4.737 (1.453, 15.445)	
	Q2	16 (10.3)	1.132 (0.557, 2.301)		2.351 (0.488, 11.334)	
	Q3	13 (7.9)	0.707 (0.330, 1.514)		1.645 (0.511, 5.294)	
	Q4	27 (14.9)	Reference		Reference	
Diuretics (−)	Q1	17 (6.2)	1.414 (0.684, 2.924)	0.618	1.152 (0.302, 4.386)	0.896
	Q2	21 (6.6)	1.631 (0.830, 3.206)		1.439 (0.432, 4.797)	
	Q3	14 (4.7)	2.015 (0.962, 4.222)		5.175 (1.726, 15.518)	
	Q4	22 (7.2)	Reference		Reference	
Diuretics (+)	Q1	17 (10.1)	2.873 (1.264, 6.531)		2.936 (0.465, 18.526)	
	Q2	6 (5.0)	1.933 (0.666, 5.604)		0.521 (0.066, 4.077)	
	Q3	13 (9.3)	0.939 (0.409, 2.158)		1.445 (0.132, 15.812)	
	Q4	16 (12.2)	Reference		Reference	
eGFR ≥ 45 mL/min/1.73 m^2^	Q1	10 (5.0)	1.627 (0.678, 3.906)	0.194	2.374 (0.245, 23.011)	0.535
	Q2	8 (3.4)	2.030 (0.837, 4.922)		7.845 (0.368, 167.103)	
	Q3	18 (6.8)	1.184 (0.575, 2.436)		8.476 (1.301, 55.226)	
	Q4	22 (7.1)	Reference		Reference	
eGFR < 45 mL/min/1.73 m^2^	Q1	26 (9.8)	1.958 (0.971, 3.950)		2.494 (0.841, 7.393)	
	Q2	18 (8.2)	1.603 (0.767, 3.352)		1.201 (0.364, 3.961)	
	Q3	11 (5.5)	1.991 (0.819, 4.840)		2.600 (0.467, 14.478)	
	Q4	18 (11.6)	Reference		Reference	
Spot urine ACR < 300 mg/gCr	Q1	17 (7.9)	2.416 (1.035, 5.644)	0.913	0.421 (0.067, 2.636)	0.593
	Q2	13 (5.4)	2.378 (1.053, 5.370)		2.595 (0.112, 59.942)	
	Q3	10 (4.4)	1.505 (0.642, 3.531)		6.738 (1.044, 43.490)	
	Q4	20 (8.8)	Reference		Reference	
Spot urine ACR ≥ 300 mg/gCr	Q1	19 (7.6)	1.542 (0.769, 3.093)		3.888 (1.208, 12.512)	
	Q2	14 (6.2)	1.339 (0.616, 2.913)		1.371 (0.313, 6.008)	
	Q3	19 (7.9)	1.182 (0.573, 2.437)		2.936 (0.793, 10.867)	
	Q4	20 (8.4)	Reference		Reference	

Models were adjusted for age, sex, Charlson comorbidity index, history of DM, medication (ACEi/ARBs, diuretics, number of antihypertensive drugs), BMI, WC, SBP, DBP, hemoglobin, albumin, fasting serum glucose, HDL-C, TG, 25(OH) vitamin D, hs-CRP levels, eGFR, spot urine ACR, and ARV. Abbreviations: CI, confidence interval; K^+^/Cr, potassium-to-creatinine ratio; CI, confidence interval; eGFR, estimated glomerular filtration rate; HR, hazard ratio; Q1, 1st quartile; Q2, 2nd quartile; Q3, 3rd quartile; Q4, 4th quartile.

## Data Availability

Not applicable.

## Supplementary Materials

The following are available online at https://www.mdpi.com/article/10.3390/nu13124443/s1, Table S1: Baseline characteristics of study participants in the quartiles by spot urine Na^+^/Cr. Table S2: Baseline characteristics of study participants in the quartiles by spot urine Na^+^/K^+^. Table S3: Cox regression analysis of urine sodium excretion for clinical outcomes. Table S4: Cox regression analysis of urine Na^+^/K^+^ for clinical outcomes. Table S5: Multivariate linear regression analysis for BPV with low urine potassium excretion excluding the subjects with CKD stage 5. Table S6: Cox regression analysis of urine potassium excretion for clinical outcomes excluding the subjects with CKD stage 5. Table S7: Multivariate linear regression analysis for BPV with low urine potassium excretion excluding the subjects with CKD stage 1. Table S8: Cox regression analysis of urine potassium excretion for clinical outcomes excluding the subjects with CKD stage 1. Table S9: Multivariate linear regression analysis for ARV with low urine potassium excretion in various subgroups. Table S10: Cox regression analysis of urine potassium excretion for all-cause mortality in various subgroups.
