# Supplementary material for: Association of Urinary Potassium Excretion with Blood Pressure Variability and Cardiovascular Outcomes in Patients with Pre-Dialysis Chronic Kidney Disease
Source: Nutrients. 2021 Dec 13;13(12):4443. doi: 10.3390/nu13124443 (PMC8706671; doi:10.3390/nu13124443)
Supplement: Supplementary file 1 [file nutrients-13-04443-s001.zip › nutrients-1432121-supplementary.pdf]

- Supplemental Materials –

Association of urinary potassium excretion with blood pressure variability and cardiovascular outcomes in patients with pre-dialysis chronic kidney disease

Sang Heon Suh, M.D., Ph.D.[1], Su Hyun Song, M.D., M.S.[1], Tae Ryom Oh, M.D., Ph.D.[1], Hong Sang Choi, M.D., Ph.D.[1], Chang Seong Kim, M.D., Ph.D.[1], Eun Hui Bae, M.D., Ph.D.[1], Kook-Hwan Oh, M.D., Ph.D.[2], Joongyub Lee, M.D., Ph.D.[3], Seung Hyeok Han, M.D, Ph.D.[4], Yeong Hoon Kim, M.D., Ph.D.[5], Dong Wan Chae, M.D., Ph.D.[6], Seong Kwon Ma*, M.D., Ph.D.[1], and Soo Wan Kim*, M.D., Ph.D.[1], on behalf of the Korean Cohort Study for Outcomes in Patients With Chronic Kidney Disease (KNOW-CKD) Investigators

[1]Department of Internal Medicine, Chonnam National University Medical School and Chonnam National University Hospital, Gwangju, Korea

[2]Department of Internal Medicine, Seoul National University, Seoul, Korea

[3]Department of Prevention and Management, School of Medicine, Inha University, Incheon, Republic of Korea

[4]Department of Internal Medicine, College of Medicine, Institute of Kidney Disease Research, Yonsei University, Seoul, Korea

[5]Department of Nephrology, College of Medicine, Inje University, Busan, Korea

[6]Department of Internal Medicine, Seoul National University Bundang Hospital, Seongnam, Gyeonggi-do, Korea

Corresponding authors

*Seong Kwon Ma, MD, PhD. Department of Internal Medicine, Chonnam National University Medical School, 42 Jebongro, Gwangju 61469, Korea, Tel: +82-62-220-6579, Fax: +82-62-225-8578, Email: drmsk@hanmail.net

*Soo Wan Kim, MD, PhD. Department of Internal Medicine, Chonnam National University Medical School, 42 Jebongro, Gwangju 61469, Korea, Tel: +82-62-220-6271, Fax: +82-62-225-8578, Email: skimw@chonnam.ac.kr

Table of Contents

Table S1. Baseline characteristics of study participants in the quartiles by spot urine Na^+/Cr

Table S2. Baseline characteristics of study participants in the quartiles by spot urine Na^+/K^+

Table S3. Cox regression analysis of urine sodium excretion for clinical outcomes

Table S4. Cox regression analysis of urine Na^+/K^+ for clinical outcomes

Table S5. Multivariate linear regression analysis for BPV with low urine potassium excretion excluding the subjects with CKD stage 5

Table S6. Cox regression analysis of urine potassium excretion for clinical outcomes excluding the subjects with CKD stage 5

Table S7. Multivariate linear regression analysis for BPV with low urine potassium excretion excluding the subjects with CKD stage 1

Table S8. Cox regression analysis of urine potassium excretion for clinical outcomes excluding the subjects with CKD stage 1

Table S9. Multivariate linear regression analysis for ARV with low urine potassium excretion in various subgroups

Table S10. Cox regression analysis of urine potassium excretion for all-cause mortality in various subgroups

Table S1. Baseline characteristics of study participants in the quartiles by spot urine Na^+/Cr

	Spot urine Na^+/Cr				
	Q1	Q 2	Q 3	Q 4	*P* value
Urine Na^+/Cr (mmol/gCr)	34.156 ± 13.115	71.943 ± 9.820	109.523 ± 11.911	189.753 ± 62.032	< 0.001
Age (year)	52.411 ± 12.714	53.378 ± 12.256	53.587 ± 11.577	55.056 ± 11.438	0.009
Male	321 (69.0)	310 (66.7)	271 (58.3)	218 (46.9)	< 0.001
Charlson comorbidity index					0.480
0 – 3	342 (73.5)	348 (74.8)	343 (73.8)	338 (72.7)	
4 – 5	114 (24.5)	109 (23.4)	119 (25.6)	118 (25.4)	
≥ 6	9 (1.9)	8 (1.7)	3 (0.6)	9 (1.9)	
History of DM	146 (31.4)	135 (29.0)	147 (31.6)	162 (34.8)	0.586
Medication					
ACEi/ARBs	381 (87.8)	418 (93.9)	393 (91.0)	409 (91.1)	0.018
Diuretics	145 (33.4)	134 (30.1)	132 (30.6)	150 (33.4)	0.585
Number of antihypertensive drugs ≥ 3	153 (32.9)	133 (28.6)	125 (26.9)	154 (33.1)	0.092
BMI (kg/m^2)	24.419 ± 3.386	24.653 ± 3.218	24.565 ± 3.493	24.623 ± 3.399	0.724
WC	86.738 ± 9.444	87.882 ± 9.537	96.920 ± 9.791	87.786 ± 9.915	0.188
SBP (mmHg)	125.800 ± 14.592	126.082 ± 14.574	127.316 ± 16.021	128.890 ± 16.180	0.008
DBP (mmHg)	76.572 ± 10.515	76.916 ± 10.212	76.869 ± 11.509	76.832 ± 11.417	0.964
Laboratory findings					
Serum K^+ (mEq/L)	4.478 ± 0.631	4.591 ± 0.636	4.565 ± 0.655	4.591 ± 0.696	0.029
24-hour urine K^+ (mEq/day)	54.269 ± 42.284	54.722 ± 24.712	51.545 ± 18.105	54.526 ± 21.649	0.613
Hemoglobin (g/dL)	13.242 ± 1.999	13.070 ± 1.951	12.937 ± 1.950	12.661 ± 1.953	< 0.001
Albumin (g/dL)	4.239 ± 0.400	4.208 ± 0.345	4.187 ± 0.375	4.191 ± 0.410	0.149
Total cholesterol (mg/dL)	170.359 ± 37.037	173.397 ± 38.337	175.562 ± 38.801	176.559 ± 37.049	0.063
HDL-C (mg/dL)	48.464 ± 15.924	49.059 ± 15.687	49.695 ± 14.816	51.206 ± 15.654	0.048
LDL-C (mg/dL)	94.889 ± 30.345	95.946 ± 30.971	96.319 ± 30.816	98.540 ± 30.240	0.324
TG (mg/dL)	156.218 ± 98.363	159.488 ± 102.875	158.821 ± 100.462	152.912 ± 93.190	0.742
Fasting glucose (mg/dL)	110.076 ± 37.749	110.108 ± 42.353	109.392 ± 36.506	110.328 ± 36.391	0.984
25(OH) Vitamin D (ng/mL)	20.078 ± 10.870	18.520 ± 8.985	18.634 ± 8.876	17.113 ± 9.809	0.003

hsCRP (mg/dL)	0.640 [0.200, 1.800]	0.600 [0.210, 1.470]	0.500 [0.200, 1.700]	0.600 [0.300, 1.600]	0.256
eGFR (mL/min./1.73m^2)	55.510 ± 31.080	52.697 ± 28.262	54.024 ± 27.949	58.086 ± 32.035	0.040
Spot urine ACR (mg/g Cr)	258.928 [67.606, 678.100]	333.802 [72.875, 1037.514]	285.232 [64.589, 923.782]	400.713 [77.290, 1021.283]	< 0.001
CKD stages					0.097
Stage 1	84 (18.1)	62 (13.3)	66 (14.2)	101 (21.7)	
Stage 2	92 (19.8)	90 (19.4)	98 (21.1)	92 (19.8)	
Stage 3a	70 (15.1)	93 (20.0)	93 (20.0)	66 (14.2)	
Stage 3b	106 (22.8)	109 (23.4)	107 (23.0)	100 (21.5)	
Stage 4	97 (20.9)	92 (19.8)	87 (18.7)	89 (19.1)	
Stage 5	16 (3.4)	19 (4.1)	14 (3.0)	17 (3.7)	

Note: Values for categorical variables are given as number (percentage); values for continuous variables, as mean ± standard deviation or median [interquartile range]. Abbreviations: ACEi, angiotensin converting enzyme inhibitor; ACR, albumin-to-creatinine ratio; ARB, angiotensin receptor blocker; BMI, body mass index; CKD, chronic kidney disease; CKD-EPI, Chronic Kidney Disease Epidemiology Collaboration; Cr, creatinine; DBP, diastolic blood pressure; DM, diabetes mellitus; eGFR, estimated glomerular filtration rate; hsCRP, high-sensitivity C-reactive protein; HTN, hypertension; K^+/Cr, potassium/creatinine ratio; SBP, blood pressure, WC, waist circumference; WHR, waist-to-hip ratio.

Table S2. Baseline characteristics of study participants in the quartiles by spot urine Na^+/K^+

	Spot urine Na^+/K^+				
	Q1	Q 2	Q 3	Q 4	*P* value
Urine Na^+/K^+	0.814 ± 0.273	1.542 ± 0.185	2.288 ± 0.249	3.943 ± 1.230	< 0.001
Age (year)	53.196 ± 12.404	54.185 ± 11.459	53.918 ± 11.332	53.135 ± 12.878	0.451
Male	274 (58.9)	275 (59.1)	275 (59.3)	296 (63.5)	0.416
Charlson comorbidity index					0.114
0 – 3	358 (77.0)	351 (75.5)	339 (73.1)	323 (69.3)	
4 – 5	104 (22.4)	105 (22.6)	115 (24.8)	136 (29.2)	
≥ 6	3 (0.6)	9 (1.9)	10 (2.1)	7 (1.5)	
History of DM	138 (29.7)	136 (29.2)	145 (31.3)	171 (36.7)	0.193
Medication					
ACEi/ARBs	382 (88.4)	410 (93.8)	411 (92.4)	398 (89.2)	
Use of diuretics	134 (31.0)	126 (28.8)	142 (31.9)	159 (35.7)	0.177
Number of antihypertensive drugs ≥ 3	140 (30.1)	122 (26.2)	132 (28.4)	171 (36.7)	0.004
BMI (kg/m^2)	24.388 ± 3.291	24.364 ± 3.329	24.833 ± 3.387	24.676 ± 3.472	0.097
WC (cm)					
SBP (mmHg)	125.555 ± 14.343	125.432 ± 14.709	127.573 ± 15.296	129.524 ± 16.804	< 0.001
DBP (mmHg)	76.529 ± 10.285	76.839 ± 10.306	76.700 ± 10.740	77.120 ± 12.252	0.866
Laboratory findings					
Serum K^+ (mEq/L)	4.455 ± 0.594	4.555 ± 0.647	4.588 ± 0.668	4.626 ± 0.700	0.001
24-hour urine K^+ (mEq/day)	60.995 ± 42.766	54.003 ± 21.368	51.857 ± 19.177	46.306 ± 18.768	< 0.000
Hemoglobin (g/dL)	13.253 ± 1.901	13.063 ± 1.921	12.997 ± 1.997	12.598 ± 2.018	< 0.001
Albumin (g/dL)	4.253 ± 0.365	4.204 ± 0.388	4.204 ± 0.366	4.164 ± 0.409	0.006
Total cholesterol (mg/dL)	173.877 ± 37.006	175.173 ± 38.262	174.335 ± 37.096	172.502 ± 39.099	0.751
HDL-C (mg/dL)	50.121 ± 16.006	50.761 ± 16.201	48.941 ± 14.065	48.597 ± 15.776	0.122
LDL-C (mg/dL)	97.054 ± 29.836	97.176 ± 31.488	95.920 ± 30.723	95.534 ± 30.391	0.805
TG (mg/dL)	153.941 ± 93.076	153.991 ± 103.415	164.493 ± 103.564	155.043 ± 94.442	0.301
Fasting glucose (mg/dL)	110.451 ± 40.739	108.857 ± 31.859	109.029 ± 39.263	111.530 ± 40.670	0.684
25(OH) Vitamin D (ng/mL)	20.197 ± 10.127	18.808 ± 9.640	19.199 ± 9.588	16.239 ± 9.078	< 0.001

hsCRP (mg/dL)	0.600 [0.200, 1.600]	0.600 [0.200, 1.400]	0.650 [0.300, 1.900]	0.600 [0.300, 1.800]	0.604
eGFR (mL/min./1.73m^2)	60.697 ± 31.465	57.133 ± 29.586	51.950 ± 28.000	50.540 ± 29.534	< 0.001
Spot urine ACR (mg/g Cr)	240.228 [44.706, 711.697]	295.643 [64.456, 865.901]	359.382 [104.279, 966.592]	383.586 [104.246, 1165.183]	< 0.001
CKD stages					0.001
Stage 1	106 (22.8)	83 (17.8)	62 (13.4)	62 (13.3)	
Stage 2	99 (21.3)	102 (21.9)	90 (19.4)	81 (17.4)	
Stage 3a	80 (17.2)	84 (18.1)	82 (17.7)	76 (16.3)	
Stage 3b	96 (20.6)	102 (21.9)	111 (23.9)	113 (24.2)	
Stage 4	69 (14.8)	78 (16.8)	105 (22.6)	113 (24.2)	
Stage 5	15 (3.2)	16 (3.4)	14 (3.0)	21 (4.5)	

Note: Values for categorical variables are given as number (percentage); values for continuous variables, as mean ± standard deviation or median [interquartile range]. Abbreviations: ACEi, angiotensin converting enzyme inhibitor; ACR, albumin-to-creatinine ratio; ARB, angiotensin receptor blocker; BMI, body mass index; CKD, chronic kidney disease; CKD-EPI, Chronic Kidney Disease Epidemiology Collaboration; Cr, creatinine; DBP, diastolic blood pressure; DM, diabetes mellitus; eGFR, estimated glomerular filtration rate; hsCRP, high-sensitivity C-reactive protein; HTN, hypertension; K^+/Cr, potassium/creatinine ratio; SBP, blood pressure, WC, waist circumference; WHR, waist-to-hip ratio.

Table S3. Cox regression analysis of urine sodium excretion for clinical outcomes

	Spot urine Na^+/Cr	Cases, n (%)	Unadjusted		Adjusted	
			HR (95%CIs)	*P* value	HR (95%CIs)	*P* value
eMACE	Q1	29 (6.2)	Reference		Reference	
	Q2	28 (6.0)	1.109 (0.621, 1.982)	0.726	1.018 (0.560, 1.850)	0.955
	Q3	32 (6.9)	1.239 (0.697, 2.203)	0.465	1.245 (0.690, 2.245)	0.467
	Q4	43 (9.2)	1.555 (0.902, 2.680)	0.112	1.340 (0.752, 2.388)	0.320
All-cause mortality	Q1	20 (4.3)	Reference		Reference	
	Q2	17 (3.7)	0.843 (0.407, 1.747)	0.646	0.925 (0.419, 2.042)	0.847
	Q3	19 (4.1)	1.063 (0.526, 2.151)	0.865	1.119 (0.523, 2.396)	0.772
	Q4	15 (3.2)	0.671 (0.308, 1.460)	0.314	0.562 (0.244, 1.296)	0.177

Note: Models were adjusted for age, sex, Charlson comorbidity index, history of DM, medication (ACEi/ARBs, diuretics, number of antihypertensive drugs), BMI, WC, SBP, DBP, hemoglobin, albumin, fasting serum glucose, HDL-C, TG, 25(OH) vitamin D, hs-CRP levels, eGFR, spot urine ACR, and ARV. Abbreviations: CI, confidence interval; CI, confidence interval; eGFR, estimated glomerular filtration rate; HR, hazard ratio; Na^+/Cr, sodium-to-creatinine ratio; Q1, 1st quartile; Q2, 2nd quartile; Q3, 3rd quartile; Q4, 4th quartile.

Table S4. Cox regression analysis of urine Na^+/K^+ for clinical outcomes

	Spot urine Na^+/K^+	Cases, n (%)	Unadjusted		Adjusted	
			HR (95%CIs)	*P* value	HR (95%CIs)	*P* value
eMACE	Q1	26 (5.6)	Reference		Reference	
	Q2	37 (8.0)	1.217 (0.693, 2.138)	0.494	1.244 (0.700, 2.211)	0.456
	Q3	30 (6.5)	1.128 (0.639, 1.992)	0.677	1.102 (0.619, 1.963)	0.742
	Q4	39 (8.4)	1.358 (0.786, 2.346)	0.273	1.197 (0.680, 2.109)	0.534
All-cause mortality	Q1	20 (4.3)	Reference		Reference	
	Q2	18 (3.9)	0.830 (0.405, 1.702)	0.612	0.790 (0.365, 1.710)	0.550
	Q3	16 (3.4)	0.694 (0.328, 1.468)	0.340	0.657 (0.301, 1.435)	0.292
	Q4	17 (3.6)	0.806 (0.393, 1.653)	0.556	0.617 (0.285, 1.335)	0.220

Note: Models were adjusted for age, sex, Charlson comorbidity index, history of DM, medication (ACEi/ARBs, diuretics, number of antihypertensive drugs), BMI, WC, SBP, DBP, hemoglobin, albumin, fasting serum glucose, HDL-C, TG, 25(OH) vitamin D, hs-CRP levels, eGFR, spot urine ACR, and ARV. Abbreviations: CI, confidence interval; CI, confidence interval; eGFR, estimated glomerular filtration rate; HR, hazard ratio; Q1, 1st quartile; Q2, 2nd quartile; Q3, 3rd quartile; Q4, 4th quartile.

Table S5. Multivariate linear regression analysis for BPV with low urine potassium excretion excluding the subjects with CKD stage 5

	Unadjusted		Adjusted	
	Coefficients (95%CIs)	*P* value	Coefficients (95%CIs)	*P* value
ARV	1.265 (0.547, 1.983)	0.001	1.092 (0.353, 1.832)	0.004
SD	0.558 (-0.032, 1.148)	0.064	0.418 (-0.194, 1.031)	0.180
CoV	0.005 (0.000, 0.009)	0.047	0.004 (-0.001, 0.009)	0.157

Note: Models were adjusted for age, sex, Charlson comorbidity index, history of DM, medication (ACEi/ARBs, diuretics, number of antihypertensive drugs), BMI, WC, SBP, DBP, hemoglobin, albumin, fasting serum glucose, HDL-C, TG, 25(OH) vitamin D, hs-CRP levels, eGFR and spot urine ACR.

Abbreviations: ARV, average real variability; CI, confidence interval; CoV, coefficient of variation; SD, standard deviation.

Table S6. Cox regression analysis of urine potassium excretion for clinical outcomes excluding the subjects with CKD stage 5

	Spot urine K^+/Cr	Cases, n (%)	Unadjusted		Adjusted	
			HR (95%CIs)	*P* value	HR (95%CIs)	*P* value
eMACE	Q1	35 (7.9)	1.907 (1.113, 3.267)	0.019	2.475 (1.128, 5.428)	0.024
	Q2	26 (5.3)	1.671 (0.954, 2.929)	0.073	1.147 (0.508, 2.591)	0.741
	Q3	29 (6.4)	1.390 (0.810, 2.383)	0.232	1.586 (0.775, 3.249)	0.207
	Q4	39 (3.6)	Reference		Reference	
All-cause mortality	Q1	16 (3.6)	0.678 (0.290, 1.586)	0.370	0.578 (0.224, 1.491)	0.257
	Q2	20 (4.5)	1.432 (0.707, 2.899)	0.319	1.200 (0.545, 2.641)	0.650
	Q3	15 (3.3)	0.908 (0.414, 1.991)	0.811	0.852 (0.375, 1.932)	0.701
	Q4	17 (3.8)	Reference		Reference	

Note: Models were adjusted for age, sex, Charlson comorbidity index, history of DM, medication (ACEi/ARBs, diuretics, number of antihypertensive drugs), BMI, WC, SBP, DBP, hemoglobin, albumin, fasting serum glucose, HDL-C, TG, 25(OH) vitamin D, hs-CRP levels, eGFR, spot urine ACR, and ARV.

Abbreviations: CI, confidence interval; K^+/Cr, potassium-to-creatinine ratio; CI, confidence interval; eGFR, estimated glomerular filtration rate; HR, hazard ratio; Q1, 1st quartile; Q2, 2nd quartile; Q3, 3rd quartile; Q4, 4th quartile.

Table S7. Multivariate linear regression analysis for BPV with low urine potassium excretion excluding the subjects with CKD stage 1

	Unadjusted		Adjusted	
	**Coefficients (95%CIs)**	***P* value**	**Coefficients (95%CIs)**	***P* value**
**ARV**	1.334 (0.575, 2.113)	0.001	1.225 (0.433, 2.018)	0.002
**SD**	0.525 (-0.096, 1.146)	0.098	0.494 (-0.150, 1.139)	0.133
**CoV**	0.005 (0.000, 0.010)	0.062	0.004 (-0.001, 0.009)	0.107

Note: Models were adjusted for age, sex, Charlson comorbidity index, history of DM, medication (ACEi/ARBs, diuretics, number of antihypertensive drugs), BMI, WC, SBP, DBP, hemoglobin, albumin, fasting serum glucose, HDL-C, TG, 25(OH) vitamin D, hs-CRP levels, eGFR and spot urine ACR. Abbreviations: ARV, average real variability; CI, confidence interval.

Table S8. Cox regression analysis of urine potassium excretion for clinical outcomes excluding the subjects with CKD stage 1

	Spot urine K^+/Cr	Cases, n (%)	Unadjusted		Adjusted	
			HR (95%CIs)	*P* value	HR (95%CIs)	*P* value
**eMACE**	Q1	34 (8.2)	1.802 (1.029, 3.158)	0.039	2.857 (1.247, 6.546)	0.013
	Q2	26 (6.5)	1.534 (0.860, 2.736)	0.147	1.293 (0.561, 2.980)	0.546
	Q3	27 (6.9)	1.150 (0.645, 2.051)	0.635	1.404 (0.619, 3.186)	0.417
	Q4	35 (10.4)	Reference		Reference	
**All-cause mortality**	Q1	17 (4.1)	0.595 (0.261, 1.358)	0.217	0.623 (0.247, 1.571)	0.316
	Q2	19 (4.7)	1.099 (0.539, 2.244)	0.795	1.171 (0.529, 2.590)	0.697
	Q3	17 (4.3)	0.879 (0.413, 1.870)	0.737	0.992 (0.448, 2.193)	0.984
	Q4	17 (5.1)	Reference		Reference	

Note: Models were adjusted for age, sex, Charlson comorbidity index, history of DM, medication (ACEi/ARBs, diuretics, number of antihypertensive drugs), BMI, WC, SBP, DBP, hemoglobin, albumin, fasting serum glucose, HDL-C, TG, 25(OH) vitamin D, hs-CRP levels, eGFR, spot urine ACR, and ARV. Abbreviations: CI, confidence interval; K^+/Cr, potassium-to-creatinine ratio; CI, confidence interval; eGFR, estimated glomerular filtration rate; HR, hazard ratio; Q1, 1st quartile; Q2, 2nd quartile; Q3, 3rd quartile; Q4, 4th quartile.

Table S9. Multivariate linear regression analysis for ARV with low urine potassium excretion in various subgroups

	Unadjusted		Adjusted	
	Coefficients (95%CIs)	*P* for interaction	Coefficients (95%CIs)	*P* for interaction
Age < 60 years	0.405 (-1.138, 1.948)	0.087	-0.569 (-2.159, 1.021)	0.247
Age ≥ 60 years	1.568 (0.566, 2.569)		1.067 (0.023, 2.112)	
Diuretics (-)	1.122 (0.273, 1.970)	0.671	0.995 (0.126, 1.864)	0.587
Diuretics (+)	1.275 (-0.054, 2.603)		1.529 (0.135, 2.923)	
eGFR ≥ 45 mL/min/1.73m^2	0.098 (-0.843, 1.039)	0.014	0.014 (-0.957, 0.985)	0.026
eGFR < 45 mL/min/1.73m^2	1.884 (0.790, 2.978)		2.116 (0.970, 3.262)	
Spot urine ACR < 300 mg/gCr	0.592 (-0.331, 1.516)	0.409	0.684 (-0.282, 1.649)	0.217
Spot urine ACR ≥ 300 mg/gCr	1.727 (0.666, 2.787)		1.695 (0.583, 2.806)	

Note: Models were adjusted for age, sex, Charlson comorbidity index, history of DM, medication (ACEi/ARBs, diuretics, number of antihypertensive drugs), BMI, WC, SBP, DBP, hemoglobin, albumin, fasting serum glucose, HDL-C, TG, 25(OH) vitamin D, hs-CRP levels, eGFR and spot urine ACR. Abbreviations: ACR, albumin-to-creatinine ratio; CI, confidence interval; eGFR, estimated glomerular filtration rate.

Table S10. Cox regression analysis of urine potassium excretion for all-cause mortality in various subgroups

	Spot urine K^+/Cr	Cases, n (%)	Unadjusted		Adjusted	
			HR (95%CIs)	*P* for interaction	HR (95%CIs)	*P* for interaction
**Age < 60 years**	Q1	6 (1.9)	1.183 (0.265, 5.286)	0.373	1.360 (0.192, 9.625)	0.708
	Q2	7 (102)	1.800 (0.450, 7.197)		5.642 (0.859, 37.064)	
	Q3	4 (1.3)	1.269 (0.284, 5.672)		2.373 (0.354, 15.928)	
	Q4	3 (1.1)	Reference		Reference	
**Age ≥ 60 years**	Q1	11 (7.7)	0.659 (0.239, 1.815)		0.417 (0.129, 1.351)	
	Q2	13 (8.4)	1.400 (0.613, 3.196)		1.057 (0.390, 2.867)	
	Q3	13 (7.9)	0.970 (0.403, 2.332)		0.820 (0.318, 2.114)	
	Q4	14 (7.7)	Reference		Reference	
**Diuretics (-)**	Q1	5 (1.8)	0.882 (0.237, 3.283)	0.814	0.619 (0.130, 2.954)	0.851
	Q2	11 (3.5)	2.139 (0.743, 6.155)		1.604 (0.470, 5.467)	
	Q3	5 (1.7)	1.008 (0.292, 3.485)		0.810 (0.214, 3.064)	
	Q4	8 (2.6)	Reference		Reference	
**Diuretics (+)**	Q1	10 (5.9)	2.873 (1.264, 6.531)		2.936 (0.465, 18.526)	
	Q2	9 (7.4)	1.933 (0.666, 5.604)		0.521 (0.066, 4.077)	
	Q3	11 (7.9)	0.939 (0.409, 2.158)		1.445 (0.132, 15.812)	
	Q4	9 (6.9)	Reference		Reference	
**eGFR ≥ 45 mL/min/1.73m^2**	Q1	3 (1.5)	0.477 (0.096, 2.366)	0.528	0.683 (0.088, 5.325)	0.989
	Q2	5 (2.1)	1.029 (0.314, 3.372)		1.964 (0.381, 10.130)	
	Q3	5 (1.9)	0.955 (0.291, 3.133)		1.613 (0.354, 7.351)	
	Q4	8 (2.6)	Reference		Reference	
**eGFR < 45 mL/min/1.73m^2**	Q1	14 (5.3)	0.635 (0.230, 1.750)		0.688 (0.208, 2.277)	
	Q2	15 (6.5)	1.343 (0.542, 3.327)		1.442 (0.497, 4.180)	
	Q3	12 (6.0)	0.947 (0.353, 2.543)		0.919 (0.315, 2.680)	
	Q4	9 (5.8)	Reference		Reference	
**Spot urine ACR < 300 mg/gCr**	Q1	8 (3.7)	0.867 (0.264, 2.842)	0.665	0.971 (0.237, 3.982)	0.703
	Q2	8 (3.3)	1.203 (0.417, 3.467)		1.093 (0.338, 3.532)	

	Q3	9 (4.0)	1.273 (0.441, 3.674)	1.053 (0.333, 3.352)
	Q4	9 (4.0)	Reference	Reference
**Spot urine ACR ≥ 300 mg/gCr**	Q1	9 (3.6)	0.638 (0.202, 2.011)	0.392 (0.062, 2.499)
	Q2	12 (5.3)	1.637 (0.634, 4.224)	1.32 (0.298, 5.959)
	Q3	8 (3.3)	0.840 (0.282, 2.499)	0.536 (0.116, 2.468)
	Q4	8 (3.4)	Reference	Reference

Note: Models were adjusted for age, sex, Charlson comorbidity index, history of DM, medication (ACEi/ARBs, diuretics, number of antihypertensive drugs), BMI, WC, SBP, DBP, hemoglobin, albumin, fasting serum glucose, HDL-C, TG, 25(OH) vitamin D, hs-CRP levels, eGFR, spot urine ACR, and ARV. Abbreviations: CI, confidence interval; DM, diabetes mellitus; K^+/Cr, potassium-to-creatinine ratio; CI, confidence interval; eGFR, estimated glomerular filtration rate; HR, hazard ratio; Q1, 1st quartile; Q2, 2nd quartile; Q3, 3rd quartile; Q4, 4th quartile.
